# Concomitant Interstitial Lung Disease with Psoriasis

**DOI:** 10.1155/2019/5919304

**Published:** 2019-08-25

**Authors:** Genta Ishikawa, Sakshi Dua, Aditi Mathur, Samuel O. Acquah, Mary Salvatore, Mary B. Beasley, Maria L. Padilla

**Affiliations:** ^1^Division of Pulmonary, Critical Care and Sleep Medicine, Icahn School of Medicine at Mount Sinai, New York, NY, USA; ^2^Department of Radiology, Columbia University Medical Center, New York, NY, USA; ^3^Department of Pathology, Icahn School of Medicine at Mount Sinai, New York, NY, USA

## Abstract

**Background:**

We encounter interstitial lung disease (ILD) patients with psoriasis. The aim of this case series was to examine clinical and radiographic characteristics of patients with concomitant psoriasis and ILD.

**Methods:**

This is a retrospective review of our institutional experience of ILD concomitant with psoriasis, from the database in the Advanced Lung/Interstitial Lung Disease Program at the Mount Sinai Hospital. Out of 447 ILD patients, we identified 21 (4.7%) with antecedent or concomitant diagnosis of psoriasis. Clinical, radiographic, pathological, and outcome data were abstracted from our medical records.

**Results:**

Median age was 66 years (range, 46–86) and 14 (66.7%) were male. Thirteen (61.9%) had not previously or concomitantly been exposed to immunosuppressive therapy directed against psoriasis. Two (9.5%) ultimately died. Clinical diagnosis of ILD included idiopathic pulmonary fibrosis, 11 (52.4%); nonspecific interstitial pneumonia (NSIP), 2 (9.5%); cryptogenic organizing pneumonia, 2 (9.5%); chronic hypersensitivity pneumonitis, 2 (9.5%); and the others, while radiographic diagnosis included usual interstitial pneumonia pattern, 9 (42.9%); NSIP pattern, 6 (28.6%); organizing pneumonia pattern, 4 (19.0%); hypersensitivity pneumonitis pattern, 2 (9.5%); and the others.

**Conclusions:**

We report 21 ILD cases with antecedent or concomitant diagnosis of psoriasis. Further prospective studies are required to determine the association between ILD and psoriasis.

## 1. Introduction

Psoriasis commonly presents with sharply defined erythematous plaques with overlying silvery scales. The scalp, elbow extensors, knees, and back are common locations for plaque psoriasis [1]. This hyperproliferative state is characterised by increased numbers of epidermal stem cells and cells undergoing DNA synthesis, shortened keratinocyte cell cycle time, and decreased epidermal turnover time [1]. Psoriatic arthritis is an inflammatory arthritis/synovitis that occurs in 25% of patients with psoriasis; it is a distinct clinical entity characterised by involvement of the distal interphalangeal joints of the hands and feet as well as the absence of rheumatoid factor [2]. Previous studies demonstrate that psoriasis is associated with diabetes mellitus [3], arterial hypertension [4], obesity [5], dyslipidaemia [6], cardiovascular disease [7], and nonalcoholic fatty liver disease [8].

Interstitial lung disease (ILD) is defined as any lung disease occurring in the parenchymal interstitium (i.e., the alveolar wall or alveolar septa) or loose-binding connective tissue (i.e., the peribronchovascular sheath, interlobular septa, or pleura) [9]. Type 17 helper T (T(H)17) cells were reported to be one of the common pathways which contribute to alveolitis and enhance cytokine production in pulmonary fibroblasts. T(H)17 cells are also the hallmark of pathogenesis in psoriasis; therefore, the coexistence of ILD and psoriasis is worth exploring on the basis of possibly shared underlying immunologic pathways. A previous small study (*N* = 50) in the Tucson VA Hospital showed that pulmonary fibrosis was not more common in a population of patients with psoriasis than would be expected in a control VA population [10]. ILD is occasionally reported in patients with psoriasis, most cases of which have been reported previously as drug-induced pneumonitis secondary to concomitant use of immunosuppressants. However, in the ILD program at Mount Sinai Hospital, we frequently encounter ILD patients with a concomitant diagnosis of psoriasis, who had been free from immunosuppressive therapy directed against psoriasis. In this pilot case series, we reported our experience with ILD in patients with psoriasis to assess their clinical features and outcomes.

## 2. Methods

### 2.1. Patients

This case series was a retrospective review of our institutional experience with ILD concomitant with psoriasis at the Advanced Lung/Interstitial Lung Disease Program at Mount Sinai Hospital, New York. The medical records of 447 consecutive patients who visited the ILD program were reviewed retrospectively. The inclusion criteria were as follows: (1) patients who visited the Advanced Lung/Interstitial Lung Disease Program from October 2009 through September 2015, (2) age ≥ 18 years old, (3) a diagnosis of ILD, and (4) diagnosed with either psoriasis or psoriatic arthritis by dermatologists. Patients who had ILD associated with sarcoidosis were excluded. Of the 447 patients who visited the ILD program, 21 (4.7%) had a diagnosis of both ILD and psoriasis. All findings on computed tomography (CT) were reviewed by a board-certified radiologist (MS), and pathological specimens were reviewed by a board-certified pathologist (MBB). All clinical and outcome data of the 21 patients were abstracted from our medical records.

The following data were extracted: age, sex, symptoms, time from diagnosis of ILD (months) prior to initial enrolment, time from diagnosis of psoriasis (years), comorbid illnesses (i.e., other autoimmune diseases, gastroesophageal reflux, and pulmonary hypertension (PH)), body mass index, smoking history, New York Heart Association (NYHA) functional class, medications, home oxygen therapy, pulmonary function test, 6-minute walking test, clinical diagnosis of ILD, CT findings, pathological results, and follow-up information. We defined both radiographic “definitive UIP pattern” and “probable UIP pattern” as “UIP pattern” in the present study. Echocardiographic evidence of possible PH was defined as estimated pulmonary artery systolic pressure >40 mmHg.

Our ILD program involves weekly multidisciplinary discussions with board-certified pulmonary physicians (AM, DS, and MLP), radiologist (MS), and pathologist (MBB), who make clinical diagnoses based on the clinical, radiographic, and pathological findings of each ILD case.

This study was approved by the Institutional Review Board of Mount Sinai Hospital (HS#: 17-01088, GCO#1: 17-2284 (0001) ISMMS, IDEATE #IRB-17-02560).

### 2.2. Statistical Methods

Descriptive analyses were performed. Continuous variables are presented as median and range, and categorical variables are presented as frequency and percentage unless stated otherwise.

## 3. Results

### 3.1. Patient Characteristics and Clinical and Pathological Presentation

The characteristics of the 21 patients with ILD and an antecedent or concomitant diagnosis of either psoriasis or psoriatic arthritis are shown in Table 1; their demographic and clinical features are shown in Table 2. Median patient age was 66 years (range, 46–86 years). There were 14 (66.7%) men and 7 women. There were 15 (71.4%) current or former smokers. The most common symptom on presentation was “cough,” followed by “dyspnea on exertion.” The median time from ILD diagnosis prior to initial enrolment was 24 months (range, 0–132 months), while the median time from psoriasis diagnosis prior to the enrolment was 2 years (range, 1–22 years). Most patients maintained relatively good performance status (NYHA I/II: 19 (90.5%)).

The clinical diagnoses after multidisciplinary discussions were as follows: idiopathic pulmonary fibrosis (IPF), 11 (52.4%); nonspecific interstitial pneumonia (NSIP), 2 (9.5%); cryptogenic organizing pneumonia, 2 (9.5%); chronic hypersensitivity pneumonitis, 2 (9.5%); and the others. The median FVC% predicted was 69 (range, 38–89), and DLco% predicted was 47 (range, 17–95). Nine (42.9%) patients had PH or possible PH diagnosed by either echocardiography or right heart catheterization. One (4.8%) patient had other concomitant autoimmune diseases (polymyositis/dermatomyositis/CREST syndrome), and 3 others (14.3%) were positive for serum markers without a definitive diagnosis of collagen vascular disease. Thirteen (61.9%) had not previously or concomitantly been exposed to immunosuppressant, whereas 8 (38.1%) had been exposed. The most common immunosuppressants included methotrexate (*n* = 4), followed by etanercept (*n* = 3). Of the 4 patients who underwent lung biopsy, including 2 who underwent video-assisted thoracoscopic surgery (VATS) and 2 who underwent transbronchial lung biopsy (TBLB), 2 patients had organizing pneumonia (OP), 1 had respiratory bronchiolitis-associated interstitial lung disease/desquamative interstitial pneumonia (RBILD/DIP) pathologic patterns, and 1 had early UIP pattern.

### 3.2. Radiographic Findings

**Figure 1:**
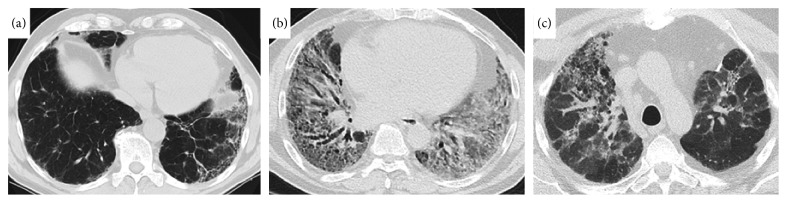
(a) Radiographic probable UIP pattern (Patient 1). (b) Radiographic NSIP pattern (Patient 11). (c) Radiographic HP pattern (Patient 10). HP: hypersensitivity pneumonitis, NSIP: nonspecific interstitial pneumonia, and UIP: usual interstitial pneumonia.

All patients had CT for current review from the time of their diagnostic evaluation. Radiographic diagnoses were as follows: usual interstitial pneumonia (UIP) pattern, 9 (42.9%, Figure 1(a)); NSIP pattern, 6 (28.6%, Figure 1(b)); organizing pneumonia pattern, 4 (19.0%); hypersensitivity pneumonitis pattern, 2 (9.5%, Figure 1(c)); and the others (Table 2). Seven patients (33.3%) had an extensive disease in CT, based on a staging system originally proposed for ILD with systemic sclerosis, by Goh et al. [11].

### 3.3. Treatments and Outcomes

Three patients with IPF started antifibrotic agents (pirfenidone, 2; nintedanib, 1) during follow-up, and 4 patients (COP, 2; IPF, 1; NSIP, 1) started additional anti-inflammatory agents (prednisone, 2; mycophenolate mofetil, 2; azathioprine, 1). During follow-up, most patients continued to have stable pulmonary function, as shown by mild worsening of predicted FVC% per year (range, −5.7% to +18%/year). One patient eventually died from pneumonia and 1 from an unknown cause (found in cardiac arrest at home).

## 4. Discussion

To our knowledge, the present case series is the first to fully investigate the clinical and radiographic features of ILD with psoriasis. Of 447 patients who visited the ILD program in a referral practice, 21 ILD patients (4.7%) had concomitant psoriasis or psoriatic arthritis, and most of them (63.6%) had not been previously or concomitantly exposed to immunosuppressants. In previous studies (Table 3), the following immunosuppressants were associated with pneumonitis in patients with psoriasis: methotrexate [32], infliximab [26], fumaric acid ester [25], leflunomide [24], gold [18], and sulfasalazine [17]. In contrast, only 3 cases of ILD without concomitant or prior use of immunosuppressants have been reported. Messina et al. [21] report a case of psoriasis with a pathologically OP pattern, even though concomitant cytomegalovirus infection might have induced pneumonitis; therefore, the causality is unclear in this case. Meanwhile, Hiki et al. report a case of severe IgA nephropathy associated with psoriatic arthritis and idiopathic interstitial pneumonia [13]. Third, Gupta and Espiritu showed a case of worsening exertional dyspnea, found to have NSIP pattern in CT chest, and subsequently developing punch biopsy-proven psoriasis [29]. Our institutional experience with 21 ILD/psoriasis cases, most of which had been without concomitant or prior use of immunosuppressants, potentially suggests a possible direct association between these 2 different disease entities. The previous small study (*N* = 50) in the Tucson VA Hospital failed to reveal their correlation, and perhaps, this might have been attributed to the underpowered nature of the study (i.e., insufficient sample size) [10]. Therefore, prospective studies with sufficient sample size, involving careful assessment with pulmonary function testing and high-resolution CT in a psoriasis cohort, are imperative for accurately determining the prevalence of ILD and assessing their possible association.

The relation between psoriasis and chronic obstructive pulmonary disease was recently demonstrated [33]. Due to its possible autoimmune-related pathogenesis, psoriasis-related immune dysfunction could lead to an abnormal immunologic response in the lung parenchyma. We hypothesise that immune dysfunction in patients with psoriasis similarly causes inflammation and fibrotic process in the lung interstitium. Activated T(H)17 cells were recently found to produce several mediators such as interleukin 17A, 17F, and 22, which induce keratinocyte proliferation and other hallmark features of psoriasis [34]. Interestingly, interleukin 17, which is produced by T(H)17 cells, contributes to alveolitis and enhances cytokine production of pulmonary fibroblasts [35]. However, the future immunology research is crucial to confirm our speculation that the pathogenic role of interleukin 17 is equivalent in the skin and lungs. Another study reports that dermal cell proliferation in psoriasis is secondary to activated TGF-*α*, which may also be associated with activated TGF-*β*, leading to pulmonary fibrosis [19]. Moreover, abnormal histologic features of lung tissue were observed in 4 (44.4%) out of 9 patients with rheumatoid factor-negative arthritis (2 with fibrosis, 1 with follicular lymphoid hyperplasia, and 1 with DIP) who did not have respiratory-related symptoms [36]. These findings collectively suggest that the coexistence of ILD and psoriasis is mechanistically plausible.

The pulmonary disease in psoriasis could potentially be similar to that in ankylosing spondylitis (AS) because of their clinical similarities (negative rheumatoid factor) between psoriatic spondylitis and AS. However, the incidence of pulmonary involvement in AS is also uncommon; a previous review at the Mayo Clinic suggests that the frequency is <2% [37]. The patients in the present study showed relatively mild impairment in FVC% predicted (median, 69; range, 38–89) in contrast to a severely decreased DLco% predicted (median, 47; range, 17–95). Given the high prevalence of concomitant PH or possible PH (42.9%) in our patients, this discrepancy between FVC and DLco might result from disproportionally elevated pulmonary artery pressure. Expectedly, mild PH is reported to be significantly more frequent in psoriasis patients, implying that increased antigen presentation, increased cutaneous T-lymphocyte activity, interleukins, and tumour necrosis factor-*α* in psoriasis cause endothelial dysfunction, which is an important pathophysiological characteristic of PH [38]. Therapeutic modalities for PH triggered by psoriasis should be investigated further.

In the present study, 11 (52.4%) patients had a clinical diagnosis of IPF; regarding radiographic findings, the UIP pattern was more common (42.9%) than the NSIP/OP pattern. This might suggest that immune dysfunction in the lungs triggered by psoriasis tends to cause fibrotic change rather than the conventional inflammatory process. The findings in our patients resemble the patterns observed in rheumatoid arthritis, in which a radiographic UIP pattern is the most common. Therefore, these 2 different diseases might have a common respiratory pathophysiology in setting of a shared clinical manifestation such as joint synovitis. Furthermore, compared to conventional IPF patients, the decline of pulmonary function was relatively mild, as indicated by a modest change in the rate of predicted FVC% (range, −5.7% to +18%/year). This also implicates a distinct feature of IPF associated with psoriasis as compared to conventional IPF. In terms of ILD treatment, Gupta and Espiritu [29] reported of a patient with NSIP associated with psoriasis responding to azathioprine, while Miyachi et al. [30] demonstrated a case which highlights the improvement of the interstitial lung pattern during psoriasis treatment with secukinumab. These two reports anecdotally support the efficacy of immunomodulatory therapy for ILD in a patient with underlying psoriasis. The efficacy of antifibrotic agents (i.e., pirfenidone and nintedanib) for psoriasis-associated IPF would be worth exploring in the future.

The major limitation of this study is that the direct association between psoriasis and ILD cannot be concluded from the findings of this case series, although the prevalence of psoriasis in our ILD cohort was not negligible. Due to the retrospective nature, the study information was loaded with several confounding factors (varied ILD patterns, smoking status, use of immunosuppressants, and other autoimmune features) that make it difficult to draw a valid conclusion. For example, the high prevalence of smoking status (71.4%) in our cohort might have been accounting for increased incidence of ILD as smoking was known to be one of the risk factors for IPF [39]. Prospective studies comparing prevalence of ILD in the psoriasis cohort and that in the general population are required to delineate the association. Second limitation is that the diagnosis of psoriasis was a self-reported diagnosis, despite being driven by dermatologists, and not based on clinical-pathological evidence saved in our electric medical record system. Likewise, most of the ILD diagnosis was only based on radiographic and clinical evaluation (except for 4 patients who underwent lung biopsies). Therefore, the inaccuracy of diagnosis makes the obtained results weaker.

## 5. Conclusion

We reported 21 ILD cases with antecedent or concomitant diagnosis of psoriasis. Among our ILD cohort, 4.7% of those had concomitant diagnosis of psoriasis or psoriatic arthritis. Considering the 3.2% prevalence of psoriasis among adults [40], this might suggest real association between two different disease entities, which cannot be concluded by this case series due to the major limitation discussed above. Further prospective and large clinical studies are warranted to investigate the association between these two different disease entities.

## Figures and Tables

**Table 1:** Patient characteristics.

	Overall *n* = 21 median (range) *n* (%)
Demographic	
Age (years)	66 (46, 86)
Male	14 (66.7)
Clinical	
Body mass index (kg/m^2^)	28.9 (20.8, 34.5)
Smoking	15 (71.4)
Home oxygen therapy	5 (23.8)
Time from diagnosis of ILD (months)	27 (0, 36)
Time from diagnosis of psoriasis (years)	2 (1, 45)
Family history of psoriasis	1 (4.8)
PH or possible PH	9 (42.9)
Gastroesophageal reflux	10 (47.6)
NYHA functional class I/II/III/IV	5/14/2/0
Pulmonary function test, % predicted	
FVC	69 (38, 89)
FEV_1.0_/FVC	80 (71, 104)
DLco	47 (17, 95)
FEV_1.0_	73 (42, 95)
6-minute walking test (m)	280 (104, 579)
Lung biopsy	5 (23.8)

ILD: interstitial lung disease; NYHA: New York Heart Association; PH: pulmonary hypertension.

**Table 2:** Demographic and clinical features of 21 ILD patients with psoriasis.

Patient	Age/sex	Arthritis	Autoimmune disease/marker	Prior/concomitant immunosuppressants	Clinical diagnosis	Radiographic diagnosis	ILD stage^*∗*^	Pathology	Outcome
1	68/M	No	SSA (+)^*∗∗*^	No	IPF	UIP pattern^#^	Limited	Not obtained	Survived at 3 months
2	66/M	No	No	No	RBILD/DIP	RBILD/DIP pattern	Limited	RBILD/DIP pattern	Survived at 34 months
3	52/M	Yes	PM/DM CREST	No	CTD-ILD	NSIP pattern	Limited	OP/EP pattern	Survived at 56 months
4	80/M	No	No	No	COP	OP pattern	Limited	OP/EP pattern	Survived at 13 months
5	78/M	No	No	No	IPF	UIP pattern	Limited	Not obtained	Survived at 51 months
6	46/M	Yes	No	MTX, etanercept	IPF/IgG4-related lung disease	NSIP/OP pattern	Limited	Not obtained	Survived at 11 months
7	58/F	Yes	No	Hydroxychloroquine sulfate, MTX, infliximab	NSIP	NSIP/OP pattern	Limited	Not obtained	Survived at 3 months
8	76/M	No	No	No	IPF	UIP pattern	Extensive	Not obtained	Unknown
9	49/M	No	No	No	IPF	UIP pattern	Limited	Not obtained	Survived at 23 months
10	60/M	No	ANCA (+)^*∗∗∗*^	No	CHP	OP/HP pattern	Extensive	Early UIP/OP	Survived at 8 months
11	62/M	No	No	No	NSIP	NSIP pattern	Extensive	Not obtained	Survived at 48 months
12	81/F	No	No	6-MP	COP	NSIP pattern	Limited	Not obtained	Died at 13 months
13	80/F	No	ANCA (+)^*∗∗∗*^	Azathioprine	IPF	UIP pattern	Limited	Not obtained	Survived at 1 month
14	63/M	No	No	No	IPF	UIP pattern	Limited	Not obtained	Survived at 46 months
15	48/M	No	No	No	IPF	NSIP pattern	Extensive	Not obtained	Survived at 18 months
16	53/F	No	No	No	Mitral valve lung disease	Reticular and GGO in lung bases with innumerable small nodules	Limited	Not obtained	Survived at 17 months
17	67/M	No	No	MTX, etanercept	IPF	UIP pattern	Extensive	Not obtained	Survived at 4 months
18	62/F	No	No	Chemotherapy for breast cancer	IPF	UIP pattern	Extensive	Not obtained	Survived at 12 months
19	86/F	No	No	No	IPF	UIP pattern	Extensive	Not obtained	Died at 8 months
20	79/M	No	No	Chemotherapy for lymphoma	Unspecific	Mosaic attenuation	Limited	Not obtained	Survived at 53 months
21	69/F	Yes	No	MTX, etanercept sulfasalazine	CHP	HP pattern	Limited	Not obtained	Survived at 4 months

ANA: antinuclear antibody, ANCA: antineutrophil cytoplasmic antibody, CHP: chronic hypersensitivity pneumonitis, COP: cryptogenic organizing pneumonia, CTD-ILD: connective tissue disease-associated interstitial lung disease, EP: eosinophilic pneumonia, F: female, GGO: ground glass opacities, HP: hypersensitivity pneumonitis, IPF: idiopathic pulmonary fibrosis, M: male, MP: mercaptpurine, MTX: methotrexate, NSIP: nonspecific interstitial pneumonia, OP: organizing pneumonia, PE: pulmonary embolus, PM/DM: polymyositis/dermatomyositis, RBILD/DIP: respiratory bronchiolitis-associated interstitial lung disease/desquamative interstitial pneumonia, SSA: SS-A antibody, and UIP: usual interstitial pneumonia. ^*∗*^A staging system, originally proposed for ILD with systemic sclerosis, by Goh et al. [11]. ^*∗∗*^Patient had a mild elevation of serum SS-A (1.20 AI) but lacked sicca symptom and other symptoms suggestive of underlying autoimmune disease (i.e., arthralgia, digital fissuring/ulceration, and Raynaud's phenomenon). ^*∗∗∗*^Patients had positivity of serum ANCA but lacked clinical symptom suggestive of underlying systemic vasculitis (i.e., skin rash, alveolar haemorrhage, and glomerulonephritis). ^#^We defined both radiological “definitive UIP pattern” and “probable UIP pattern” as “UIP pattern” in the present study.

**Table 3:** Summary of previous reports on interstitial lung disease in conjunction with psoriasis.

Reference	Age/sex	Arthritis	Concomitant autoimmune disease	Prior or concomitant immunosuppressant use	Radiological finding	Pathology
Kaplan and Waite [12]	70/M	No	No	MTX	Interstitial fibrosis with honeycombing	Not specified

Guzman [10]	54/M	Yes	No	A variety of anti-inflammatory drugs	Considerable loss of volume in both upper lobes with advanced fibrosis	Notable interstitial fibrosis with dilated bronchi and bronchioles

Hiki et al. [13]	23/M	Yes	IgA nephropathy	No	A reticular or reticulonodular pattern with bilateral pneumonic consolidation	Severe interstitial pneumonia

Salaffi et al. [14]	62/M	Yes	No	Gold salts, etretinate, sulfasalazine, MTX	Bilateral and diffuse interstitial infiltrates, broader in the upper right lobe	Not specified

Kawakami et al. [15]	50/F	No	Polymyalgia rheumatic	Not specified	Bilateral basilar reticulonodular shadows	NSIP

Ameen et al. [16]	53/F	No	No	MTX, cyclosporin	Widespread changes in the mid- and upper zones with thickening and nodularity of the interlobular septae	Not specified

Woltsche et al. [17]	78/M	Yes	No	Sulfasalazine	Small nodular densities over both lungs	Lymphoplasmocytic interstitial infiltration

Manero Ruiz et al. [18]	Not specified	Yes	No	Gold salts	Not specified	Not specified

Tokunaga et al. [19]	56/M	No	No	*Sho-saiko-to* (Chinese herbs)	Microcystic lesions, reticular shadows, and traction bronchiectasis underneath the pleura at the back of both lower lobes	UIP

Abou-Samra et al. [20]	35/F	No	No	MTX, cyclosporin, acitretin	Bilateral interstitial infiltrate and alveolar filling of the right pulmonary base	Not specified

Abou-Samra et al. [20]	61/F	No	No	Acitretin, MTX	Bilateral interstitial infiltrate of the pulmonary bases	Not specified

Messina et al. [21]	63/F	No	No	No	Diffuse interstitial infiltrates	Obliteration of the alveolar spaces connective tissue

El-Hag et al. [22]	Not specified	No	No	Infliximab, azathioprine	Not specified	Not specified

Penizzotto et al. [23]	51/F	No	No	Not specified	Not specified	Not specified

Lee and Hutchinson [24]	52/F	Yes	No	MTX, leflunomide, sulfasalazine	Ground glass appearance throughout the lung fields	Not specified

Deegan et al. [25]	49/M	No	No	Fumaric acid esters	Bilateral, mostly peripheral foci of consolidation with air bronchograms	Pattern of organizing pneumonia

Leger et al. [26]	68/F	No	No	Infliximab	Bilateral basal interstitial infiltrates with pleural effusion	Not specified

Bale and Chee [27]	67/M	No	No	Infliximab	Patchy alveolar and ground glass infiltrates	Not specified
Kakavas et al. [28]	64/M	No	No	Infliximab	Bilateral ground glass and interstitial infiltrates	Not specified

Gupta and Espiritu [29]	43/F	No	No	No	NSIP pattern	Not specified

Miyachi et al. [30]	71/F	No	No	Etretinate, cyclosporin, methotrexate	Basal interstitial ground glass opacities	Not specified

Deng et al. [31]	30/F	Yes	Rheumatoid arthritis	Prednisone, iguratimod, total glucosides of peony	Not specified	Not specified

F: female, M: male, MTX: methotrexate, NSIP: nonspecific interstitial pneumonia, and UIP: usual interstitial pneumonia.

## Data Availability

The data used to support the findings of this study are available from the corresponding author upon request.

## Abbreviations

ANA: Antinuclear antibody
ANCA: Antineutrophil cytoplasmic antibody
AS: Ankylosing spondylitis
CHP: Chronic hypersensitivity pneumonitis
CT: Computed tomography
CTD-ILD: Connective tissue disease-associated interstitial lung disease
EP: Eosinophilic pneumonia
GGO: Ground glass opacities
HP: Hypersensitivity pneumonitis
ILD: Interstitial lung disease
IPF: Idiopathic pulmonary fibrosis
MP: Mercaptpurine
MTX: Methotrexate
NSIP: Nonspecific interstitial pneumonia
NYHA: New York Heart Association
OP: Organizing pneumonia
PE: Pulmonary embolus
PM/DM: Polymyositis/dermatomyositis
PH: Pulmonary hypertension
RBILD/DIP: Respiratory bronchiolitis-associated interstitial lung disease/desquamative interstitial pneumonia
TBLB: Transbronchial lung biopsy
T(H)17: Type 17 helper T
UIP: Usual interstitial pneumonia
VATS: Video-assisted thoracoscopic surgery.
